# Enhancement of the Antioxidant and Skin Permeation Properties of Betulin and Its Derivatives

**DOI:** 10.3390/molecules26113435

**Published:** 2021-06-05

**Authors:** Andrzej Günther, Edyta Makuch, Anna Nowak, Wiktoria Duchnik, Łukasz Kucharski, Robert Pełech, Adam Klimowicz

**Affiliations:** 1 Department of Chemical Organic Technology and Polymeric Materials, Faculty of Chemical Technology and Engineering, West Pomeranian University of Technology, Szczecin, PL-70322 Szczecin, Poland; emakuch@zut.edu.pl (E.M.); rpelech@zut.edu.pl (R.P.); 2 Department of Cosmetic and Pharmaceutical Chemistry, Pomeranian Medical University in Szczecin, PL-70111 Szczecin, Poland; anowak@pum.edu.pl (A.N.); wduchnik@pum.edu.pl (W.D.); lukasz.kucharski@pum.edu.pl (Ł.K.); adam.klimowicz@pum.edu.pl (A.K.)

**Keywords:** synthesis of new betulin derivatives, antioxidant activity, chemistry of natural compounds, vehicle effect, skin barrier, stratum corneum

## Abstract

This study investigated the antioxidant activity DPPH, ABTS, and Folin–Ciocalteu methods of betulin (compound **1**) and its derivatives (compounds **2**–**11**). Skin permeability and accumulation associated with compounds 1 and **8** were also examined. Identification of the obtained products (compound **2**–**11**) and betulin isolated from plant material was based on the analysis of ^1^H- NMR and ^13^C-NMR spectra. The partition coefficient was calculated to determine the lipophilicity of all compounds. In the next stage, the penetration through pig skin and its accumulation in the skin were evaluated of ethanol vehicles containing compound **8** (at a concentration of 0.226 mmol/dm^3^), which was characterized by the highest antioxidant activity. For comparison, penetration studies of betulin itself were also carried out. Poor solubility and the bioavailability of pure compounds are major constraints in combination therapy. However, we observed that the ethanol vehicle was an enhancer of skin permeation for both the initial betulin and compound **8**. The betulin **8** derivative showed increased permeability through biological membranes compared to the parent betulin. The paper presents the transformation of polycyclic compounds to produce novel derivatives with marked antioxidant activities and as valuable intermediates for the pharmaceutical industry. Moreover, the compounds contained in the vehicles, due to their mechanism of action, can have a beneficial effect on the balance between oxidants and antioxidants in the body, minimizing the effects of oxidative stress. The results of this work may contribute to knowledge regarding vehicles with antioxidant potential. The use of vehicles for this type of research is therefore justified.

## 1. Introduction

The skin has been investigated as a route to deliver active substances contained topically in cosmetics and pharmaceuticals. Transdermal delivery of active substances is often limited by their poor permeability [1,2,3,4,5,6,7,8,9]. The stratum corneum (SC) acts as a barrier for the active substance permeation into the skin. The SC is 15–20 μm thick and is composed of 15–20 flattened layers, and the epidermal cell membranes are so tightly joined that there is hardly any intercellular space through which molecules penetrate [10,11,12]. However, transdermal delivery of active substances offers the advantages of having a relatively large absorption surface area and being non-invasive [10]. The permeation of the active substance is highly dependent on the lipophilicity and molecular size of these substances [13]. Overcoming the lipophilic barrier, which is the skin, is possible for compounds with molecular weights of <600 Da [13]. The optimal partition coefficient value (*log* P), which is an indicator of the lipophilicity of the active substance, ranges from 2 to 3 [13].

**Figure 1:**
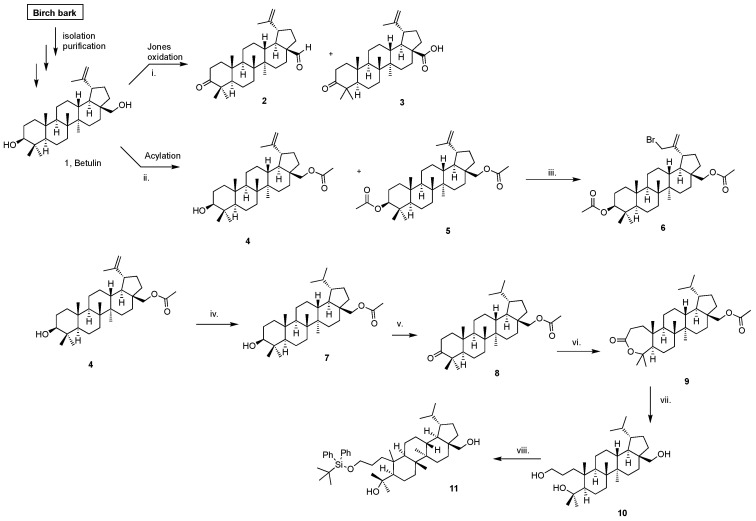
Synthesis of betulin derivatives. Reagent and conditions: i. Jones oxidation, CrO_3_, aq. H_2_SO_4_, acetone, 0 °C, (N_2_ atm); ii. DMAP, pyridine, Ac_2_O, 0 °C; iii.NBS, CCl_4_; iv. MeOH/THF, 10% Pd/C, H_2_ (40 psi); v. DCM, PCC, 0 °C; vi. chloroform, 70%-mCPBA, 35 °C; vii. THF, LiAlH_4_, −40 °C; viii. imidazole, THF, TBDPSCl, −70 °C.

A promising alternative to antioxidant agents is a natural compound, such as betulin, a component of the triterpenes synthesized by plants [14,15]. Betulin (IUPAC name lup-20(29)-en-3β,28β-diol), belonging to the lupane-type pentacyclic triterpenes is characterized by relatively high lipophilicity (partition coefficient value (*log* P) = 5.34 [16] and unfortunately it is practically insoluble in aqueous media [17,18]. Therefore, betulin has poor penetration through the skin. However, betulin and its derivatives have a broad spectrum of biological relevance, thus they have a high potential for application in transdermal systems [19,20]. To date, several formulations containing betulin and its derivatives have been developed [21,22,23,24,25]. However, there are no literature reports describing the penetration of vehicles containing these compounds through the skin. Betulin shows a broad spectrum of biological activity (including cytotoxic activity [19]) because its structure contains substituents and groups, which can be easily functionalized (such as the isopropenyl moiety, alkyl group, and hydroxyl groups) [14,15,16,26,27,28,29,30]. Therefore, it seemed interesting to obtain new betulin derivatives, especially ketones—compound **8** (Figure 1). This allows for the constant search for new substances with antioxidant properties.

This study proposes ethanol as a vehicle for betulin and its derivative for cutaneous applications. The active substance delivery platforms that have attracted the attention of specialists in the medical and pharmaceutical industries are transdermal drug delivery systems in the form of alcohols (especially ethanol) as vehicles. The main advantage of these vehicles is their approval for use in medical, pharmaceutical, and cosmetics preparations, high enhancing activity. and good solvating ability—which are promising candidates for betulin and its derivatives as antioxidants supply [4].

This study presents the effect of ethanol as a vehicle on skin permeation of compounds with antioxidant activity: betulin and its derivative **8**. The use of ethanol application in this study was to evaluate this vehicle on the permeability of the tested compounds. The pH value of the acceptor phase in in vitro permeation tests was set at 7.4 for the simulation of the skin surface [13,31,32,33]. The penetration through pig skin using the Franz diffusion cell and the accumulation of betulin and compound **8** in the skin was evaluated. Due to their mechanism of action, the compounds contained in vehicles can beneficially affect the balance between oxidants and antioxidants in the body, minimizing the effects of oxidative stress. The results of this work may contribute to knowledge regarding vehicles with antioxidant potential. The use of vehicles for this type of research is therefore justified. Our data suggest that ethanol is a promising vehicle for betulin and compound **8**.

## 2. Results

### 2.1. Betulin and Its Derivatives

Figure 1 shows the scheme of the synthesis of betulin derivatives.

Table 1 presents the partition coefficient values calculated in the MestReNova program. The lipophilicity of each compound is represented as the decimal logarithm of the partition coefficient (*log* P) [34].

The high lipophilicity (*log* P 4.777–9.684) of the obtained compounds **1**–**11**, favors their excessive accumulation in lipid membranes. This way, the diffusion of active substances administered epidermally, will be hindered through the living layers of the epidermis [34,35].

**Figure 2:**
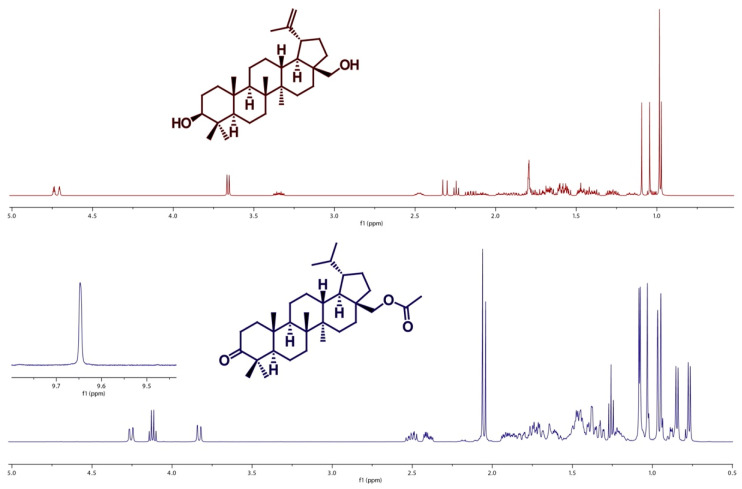
^1^H-NMR spectrum of betulin (above), and its derivative (below).

The presence of clear peaks in the ^1^H-NMR spectrum of betulin (above) and its derivative (below) indicate the presence of certain groups of protons in the structure of the tested compounds—Figure 2. (Supplementary data present ^1^H NMR and ^13^C NMR spectra of compounds **1**–**11**).

The proton signals recorded, corresponding to the hydrogen atoms from the groups belonging to the hydrocarbon chains of the compounds **1**–**11**, were obtained. The analysis of the chemical shift values of carbon atoms additionally supplemented the information and confirmed the structure of the obtained compounds. 

### 2.2. Evaluation of Free Radical Scavenging Activity

Table 2 presents the results for the antioxidant activity of betulin and its derivative, carried out using the DPPH, ABTS, and Folin–Ciocalteu methods.

The test results, presented in Table 2, show that the highest antioxidant activity (for DPPH and ABTS methods) was observed for compound **8**: 61.83 ± 0.004% RSA (for the DPPH method) and 12.79 ± 0.005% RSA (in the case of the ABTS method). The study of DPPH radical scavenging capacity of the compounds **2**, **3**, **6**, **7**, **9**, **10**, and **11** was characterized by the DPPH radical scavenging degree of: 1.50 ± 0.007% RSA, 7.04 ± 0.007% RSA, 14.78 ± 0.014% RSA, 2.80 ± 0.012% RSA, 14.11 ± 0.013% RSA, 4.84 ± 0.011% RSA, and 12.68 ± 0.013% RSA, respectively. Pure betulin was characterized by antioxidant activity 2.81 ± 0.007% RSA, while in the case of the studies carried out for compounds **4** and **5**, no antioxidant activity was shown.

The antioxidant activity (determined by the ABTS method) of the compounds obtained varied as follows: 12.79 ± 0.005% RSA (for compound **8**) >5.65 ± 0.015% RSA (for compound **11**). Pure betulin, compounds **2**–**7** and **9**–**10**, showed no antioxidant activity by this method.

In contrast, the antioxidant activity obtained by the Folin–Ciocalteu method changed as follows: 0.847 ± 0.037 mmol TE/dm^3^ (for compound **3**) >0.413 ± 0.015 mmol TE/dm^3^ (for compound **9**) >0.367 ± 0.081 mmol of TE/dm^3^ (for compound **6**) >0.050 ± 0.000 mmol of TE/dm^3^ (for compound **8**). No antioxidant activity was obtained for compounds **1**, **2**, **4**, **5**, **7**, **10**, and **11**.

### 2.3. Skin Penetration and Skin Extraction

Compound **8** is characterized by the highest degree of free radical scavenging DPPH, therefore the penetration through pig skin and its accumulation in the skin were evaluated. For comparison, penetration studies of betulin were also carried out.

HPLC analysis of the compounds present in the acceptor fluid during the 24 h experiment showed that the test compounds could still be detected in the acceptor chamber after the test period.

**Figure 3:**
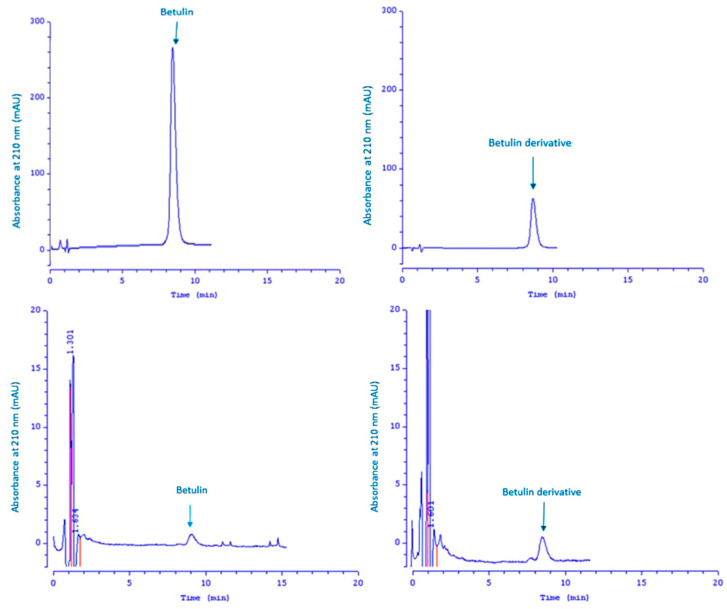
HPLC chromatograms of reference betulin and reference compound **8** (above), HPLC chromatograms of betulin and betulin derivative **8** in the acceptor fluid after a 24 h experiment (below).

For confirmation, the chromatogram of betulin and its derivative is shown—Figure 3. The left side of Figure 3 shows the chromatogram for the signal with a retention time of 9.01 min (compound **1**), while the right side of this figure shows the chromatogram for the signal with a retention time of 8.99 min (compound **8**).

The remaining peaks visible in these chromatograms come from the test mixture and are also detected in the blank. As can be seen, the obtained signals with a retention time of 9.01 (left side of this Figure) and 8.99 (right side of this Figure) are identical, therefore the analyzed compounds which penetrated the skin are betulin and its derivative **8**.

Table 3 presents the results of the permeation and the accumulation studies of the tested compounds.

**Figure 4:**
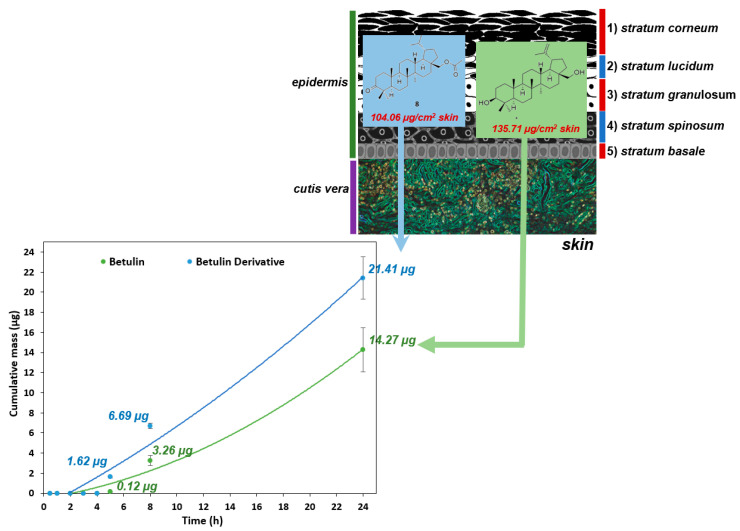
The cumulative mass of betulin and derivative **8** penetrated into the acceptor fluid during the 24 h experiment.

Figure 4 shows the comparison of the in vitro permeation profiles for betulin and derivative **8** through the skin during the 24 h experiment (Supplementary data present the skin permeation of betulin and its derivative **8**).

The permeation of the vehicle containing betulin and its derivative **8** through the pig skin was assessed, in which the donor phase consisted of the vehicles tested, moreover, the acceptor phase was the PBS solution, as it corresponds to systemic conditions [13,31,33]. As shown in Table 3, the application of compound **8** in vehicles led to an increase in its permeation through the skin in comparison to betulin applied in the same vehicle. After experimenting for 24 h, the highest mean cumulative mass was observed for compound **8** (21.41 ± 2.10 µg). The mass was slightly lower of betulin (14.27 ± 2.20 µg). The cumulative mass of betulin which penetrated into the acceptor fluid was observed only after 5 h of the experiment, while for derivative **8** after 4 h. Moreover, the average cumulative masses for vehicles containing the tested compounds at 0.5; 1; 2; 3; 4; 5; 8, and 24 h are shown in Figure 4 (Table 3).

After the experiment was carried out, the skin was extracted to evaluate the amount of the accumulated tested active ingredients. The concentrations of betulin and its derivative **8** contained in the vehicles were respectively: 135.71 ± 9.11 µg/cm^2^ for the skin, and 104.06 ± 15.79 µg/cm^2^ for the skin—Table 3.

## 3. Discussion

Betulin is a pentacyclic triterpene. Pentacyclic triterpenoids (including pentacyclic triterpenes such as betulin) are of interest to researchers due to their anti-inflammatory, antimicrobial, antitumor, cytotoxic, and other activities [14,15,18,25,27,28,29,30,36,37,38,39], and their possible use as intermediates in the synthesis of new pharmacological substances.

These compounds thus have high potential for application in transdermal systems, however, they are highly hydrophobic (poor skin penetration), which significantly limits their use as effective pharmacological agents [18]. The lipophilic parameter of betulin and its derivatives (Table 1) depends on the type of substituent in C-3 and C-28 positions (Figure 1), polar surface, polarizability, molecular volume, and molecular refraction [34,36,37,38]. In the group of tested compounds, compound **3** showed the lowest lipophilicity parameter (*log* P = 4.777). Modification of betulin by introducing the following moieties: an acyl group in position C-28 in the case of compound **4**, acyl groups in position C-3 and C-28 in the case of compound **5**, insertion of a bromine atom in the allyl position in the case of compound **6**, hydrogenation of the allyl group in the case of compound **7**, synthesis of a ketone in the C-3 position in the case of compound **8**, formation of a lactam from the A ring in the case of compound **9**, blocked –OH group by *tert*-butyldiphenylsilyl group (TBDPS) in the case of compound 11, led to an increase in lipophilicity the following compounds **4**–**9** and **11**, resulting in *log* P in the range of 6.304–9.684. Compounds: **2** (due to the introduction of a ketone group at the C-3 position and an aldehyde group at the C-28 position) and **3** (due to the introduction of a ketone group at the C3 position and a carbonyl acid at the C-28 position) were characterized by a lower *log* P value in relation to the initial betulin.

In recent years an efficient formulation has been presented containing betulin and its derivative, to enhance the delivery of these substances via different routes: betulinic acid (BA) loaded polylac-tide-co-glycolide-monomethoxy polyethylene glycol nanoparticles (PLGA-mPEG NPs), inhalable lactose based nanosystems of betulin delivery, a liposome formulation of BA (oral administration of this formulation slowed down tumor growth in mice) mulauer, copolymers (which can be used as carriers in drug delivery systems in the form of microspheres etc.), but there is still room for improvement [17,21,22,23,24,39].

Currently, one of the most common ways to increase the effectiveness and bioavailability of highly hydrophobic compounds that penetrate poorly through the skin is the application of these compounds in vehicles (in the form of alcohols—ethanol, *n*-propanol, isopropyl alcohol, *n*-butanol, *n*-pentanol, and propylene glycol [33,39,40].

The main benefit of ethanol vehicles is the approval for use in medical, pharmaceutical, and cosmetics preparations, high enhancing activity, and good solvation ability [4,13]. In addition, the good permeability of vehicle containing compound **8** through the skin and its proper accumulation in the skin (Table 3, Figure 4) as well as the high antioxidant capacity of the initial compound **8** (Table 2) may also limit the effects of free radicals, which are highly toxic molecules due to the presence of one or more unpaired electrons. Compound **8** has a ketone group in the C-3 position, which may increase the reactivity of this compound with the DPPH radical [4,13,41,42,43,44,45,46].

Our previous studies on the effect of vehicles on skin permeability of a new eugenol derivative (eugenyl dichloroacetate—EDChA) with antioxidant activity have shown that the application of a eugenol (E) derivative of the cream and gel as vehicles did not lead to an increase in the skin permeation of EDChA, in comparison to the initial E applied in the same vehicles [47].

However, the highest permeation rate of cream vehicles containing E and EDChA into the acceptor fluid (µg/h) was observed between 4 and 5 h, while for gel vehicles between 3 and 4 h [47]. In addition, after skin extraction, the obtained results showed that the concentration of substances contained in the cream vehicles decreased in the following order: eugenyl dichloroacetate > eugenol. Moreover, the concentration of substances contained in the gel vehicles decreased in the following order: E > EDChA [47].

## 4. Materials and Methods

### 4.1. Chemicals

The following compounds were used for the preparation of betulin, and the synthesis of its derivatives: 4-dimethylaminopyridine (DMAP), acetic anhydride (Ac_2_O), carbon tetrachloride (CCl_4_), tetrahydrofuran (THF), dichloromethane (DCM), palladium on carbon (10% Pd/C), pyridinium chlorochromate (PCC), 3-chloroperbenzoic acid (mCPBA), lithium aluminum hydride (LiAlH_4_), *tert*-butyl(chloro)diphenylsilane (TBDPSCl), purchased from Sigma Aldrich (Steinheim am Albuch, Germany), KOH, hexane, methanol, ethyl acetate, acetone, MgSO_4_, ethanol, purchased from Chempur (Piekary Śląskie, Poland). All reagents were of analytical grade.

For the determination of the antioxidant activity and to assess the skin permeation of the compounds obtained: acetonitrile for HPLC was from J.T. Baker (Berlin, Germany). 2,2-diphenyl-1-picrylhydrazyl (DPPH), 2,2′-azino-bis(3-ethylbenzothiazoline-6-sulfonic acid) (ABTS) and 6-hydroxy-2,5,7,8-tetramethylchroman-2-carboxylic acid (TE), were purchased from Sigma Aldrich (St. Louis, MO, USA); Folin–Ciocalteu reagent and gallic acid (GA), were from Merck (Darmstadt, Germany), potassium persulfate and chloride, sodium chloride, methanol, ethanol, acetone and phosphate buffered saline (PBS, pH = 7.4) were purchased from Chempur (Piekary Śląskie, Poland). All reagents were of analytical grade.

### 4.2. Plant Material

The plant material (the papilionaceous birch bark) was collected in June in Poland in the year 2019 (N53° 20′12”, E15° 02′59”) from the natural state, near-located places. Ten samples were harvested and combined into one collective sample.

Samples were deposited in the plant material storage room (No. BC-2019/015) at the Faculty of Chemical Technology and Engineering, Department of Chemical Organic Technology and Polymeric Materials, West Pomeranian University of Technology, Szczecin. The deposited material consisted of samples of dried papilionaceous birch bark (*Betula pendula Roth*).

### 4.3. Preparation of Betulin and Characterization of Betulin and Its Derivatives

The betulin and novel compounds were obtained in high yields and were identified by ^1^H and ^13^C-NMR, moreover, the partition coefficient for all compounds was calculated in the MestReNova program (Figure 1, Table 1).

The structures of betulin and the obtained compounds were confirmed based on the analysis of nuclear magnetic resonance (NMR) spectroscopy spectra. Measurements were performed using a Bruker DPX-400 spectrometer. ^1^H-NMR spectra were recorded under the following conditions: 400.13 MHz, a 12 kHz spectrum width, 65.5 K data points, a 0.488 Hz/point resolution, a data acquisition time of 4.09 s, a repetition time of 1 s, a 7.8 μs pulse width, and 16–32 scans. ^13^C-NMR spectra were as follows: 100.62 MHz, a spectrum width of 24 kHz, 65.5 K data points, a resolution of 1.46 Hz/point, a data acquisition time of 1.37 s, a repetition time of 1 s, a pulse width of 9.2 μs, and 1–8 scans.

#### 4.3.1. Preparation of the Birch Bark and Isolation of Betulin from the Birch Bark

The bark was boiled in a 1% aqueous KOH solution for 2 h. The bark was then filtered on a Büchner funnel and washed with hot water. The bark was dried in a vacuum dryer at 80 ℃ for 2 days.

The isolation of betulin was carried out from the shredder birch bark of *the Betula verrusoca* tree. An amount of 100 g of dried bark was first extracted with hexane (500 mL) in a Soxhlet apparatus, for 24 h (2 × 12 h), then with methylene chloride (500 mL) for 24 h (2 × 12 h). After evaporation to dryness, the hexane extract yielded 3.25 g of dry extract, while the methylene chloride extract yielded 11.17 g of dry extract.

The 11.17 g of dry extract obtained was heated under a reflux condenser in methanolic KOH 2% solution (100 mL) for 10 h. The flask was allowed to stand overnight at room temperature. The precipitate was drained on a Büchner funnel and washed copiously with hot water. The obtained impure betulin (8.4 g) was dried in a vacuum oven at 80 °C overnight. The betulin was then purified on a chromatography column in the system hexane:ethyl acetate (H:OAc) 40:1→5:1 *v*/*v*, monitoring the extraction with TLC (H:OAc 5:1). Finally 7.23 g of pure betulin was obtained (**1**).

#### 4.3.2. Synthesis of Betulonic Aldehyde (2) and Betulonic Acid (3)

An amount of 1 g (2.26 mmol) of betulin (**1**) was placed in 3-neck round-bottomed flask, in 150 mL of acetone, at 0 °C (ice bath) under N_2_. Freshly prepared Jones reagent (3.9 mL, 0.39 M, 7.68 mmol) was added dropwise from an additional funnel to the stirred suspension of betulin (**1**) over 15 min. After 1 h, stirring was continued at room temperature for 2 h, then 25 mL of methanol was added until a dark green color appeared. The reaction mixture was then poured onto 100 mL of water with ice. Methanol and acetone were removed under reduced pressure. The aqueous residue was washed 3 times with 40 mL of ethyl acetate. The combined organic layers were first washed with brine (20 mL) then with water (20 mL) and finally dried with anhydrous MgSO4. The solvent was evaporated under reduced pressure to give a crude white solid, which was purified by column chromatography to deliver first 59.7 mg (6%) of betulonal as a white solid, then 860 mg (86%) of betulonic acid as a white solid.

#### 4.3.3. Synthesis of Compounds **4** and **5** by Acetylation of Betulin

An amount of 5 g (11.3 mmol) of betulin (**1**) and DMAP (3.05 g, 25 mmol) were placed in a round-bottomed flask, in 200 mL of dry pyridine, at 0 °C (cooling bath). Then, 24.5 mL (0.25 mmol) of Ac_2_O was slowly added dropwise (over 1 h) to the stirred solution from the additional funnel. The solvent was evaporated under reduced pressure to give a crude dark solid, which was purified by column chromatography in the system hexane:ethyl acetate (H:OAc) 40:1→5:1 *v/v*, to deliver first 0.55 g (11%) of betulin 3,28-diacetate **3** as a w white crystalline powder, then 1.86 g (37%) of betulin 28-acetate as a white crystalline powder.

#### 4.3.4. Synthesis of Compound **6** (30-bromolup-20(29)-ene-3β,28-diyl diacetate)

A solution of **5** (0.5 mmol, 265 mg) in dry CCl_4_ (5 mL) was treated with freshly recrystallized NBS (0.15 g, 0.85 mmol,) and refluxed for 3 h. The solid was filtered off and the mother liquor was evaporated under vacuum. The dark solid was recrystallized from EtOH to obtain 0.183 g (69%) as brown needles.

#### 4.3.5. Synthesis of Compound **7** (3β,28-lupanediol 28-acetate)

To a solution of **4** (3.09 mmol, 1.5 g) in 60 mL mixture of MeOH/THF (2:1 *v/v*), 10% Pd/C (167 mg) was added and hydrogenated under H_2_ (40 psi) for 24 h. Next, it was filtered, and the solvent was evaporated under reduced pressure to give 1.49 g, (99+%) as a white crystalline solid.

#### 4.3.6. Synthesis of Compounds **8** (28-acetoxy-3-lupanone)

To a cooled (ice bath) solution of monoacetate **7** (1.40 g, 2.88 mmol) in 50 mL of DCM, PCC (3.68 g, 17.07 mmol) was slowly added in small portions over 5 min. The reaction was monitored by TLC. After 4 h, the flask with the reaction mixture was placed in the freezer overnight. The solvent was evaporated under reduced pressure and the obtained residue was purified by column chromatography (H:OAc 1:1 *v*/*v*) to give 1.30 g (92%) of the ketone **8** as a white solid.

#### 4.3.7. Synthesis of Compound **9**

To a solution of ketone **8** (1.20 g, 2.48 mmol) in 50 mL of chloroform was added 70%-mCPBA (3.23 g, 15 mmol). The reaction mixture was heated to 30–35 °C and after 4 h another portion of 70%-mCPBA (0.91 g, 4.22 mmol) was added. The reaction was monitored by TLC, there was still presence of the substrate. After 24 h (the substrate was still present on TLC), another portion of 70%-mCPBA (0.58 g, 2.69 mmol) was added. After 12 h (there was only a trace of the substrate, TLC), 30 mL solution of 10%-Na_2_S_2_O_3_ was slowly added (the temperature of the mixture increases). The mixture was transferred to a separatory funnel and the organic layer was washed with 30 mL of saturated NaHCO_3_ solution. The solvent was evaporated under reduced pressure and the obtained residue was purified by column chromatography (H:OAc 20:1→3:1 *v*/*v*) to give 1.06 g (85%) of **9** as a white solid.

#### 4.3.8. Synthesis of Compound **10**

To a cooled 20 mL amount of THF (about −40 °C acetonitrile with dry ice), LiAlH_4_ (606 mg, 16 mmol) was added followed by **9** (800 mg, 1.6 mmol). The reaction was stirred for 1.5 h until the substrate disappeared (TLC). Once the reaction was complete, 10 mL of ethyl acetate was added followed by slowly added 30 mL of saturated NH_4_Cl solution (until bubbling disappeared). The organic layer was separated and filtered through a thin layer of silica gel. The aqueous layer was extracted with 20 mL of ethyl acetate. The solvents were evaporated under reduced pressure and the obtained residue was purified by column chromatography (H:OAc 1:1 *v/v*) to give 736 mg (96%) of **10** as a white solid.

#### 4.3.9. Synthesis of Compound **11**

To a cooled solution (about −70 °C isopropyl alcohol and dry ice) of 10 (500 mg, 1.08 mmol) in 20 mL THF was added imidazole (235.39 mg, 3.46 mmol) and TBDPSCl (445.48 mg, 1.62 mmol, d = 1.057 g/mL). When the temperature reached ambient temperature, the mixture was further stirred for 24 h. Next, the solvent was evaporated under reduced pressure and the obtained oily residue was purified by column chromatography (H:OAc 20:1→5:1 *v*/*v*) to give 370 mg (49%) of ketone **11** as a light straw-colored oil.

### 4.4. Evaluation of Free Radical Scavenging Activity

The studies on the antioxidant activity of betulin and its derivatives were carried out using the DPPH [13,47,48], ABTS [13,47] and the Folin–Ciocalteu [13,47,49] methods. The absorbance at λ 517 nm (in the case of the DPPH method), 734 nm (in the case of the ABTS method), and 765 nm (in the case of the Folin–Ciocalteu method) working solutions was adjusted to 1.00 ± 0.02. Measurements were taken using a Spectroquant, model Pharo 300 (Merck, Germany), in triplicate for each tested sample. The efficiency of neutralization of DPPH radicals by betulin and its derivatives was expressed in the form of TEAC (Trolox Equivalent Antioxidant Capacity) factor, determining the concentration of Trolox with identical antioxidant capacity. TEAC values were calculated from absorbance measurements using the calibration curve method. The results were expressed as TEAC in millimoles of trolox per volume of sample (mmol TE/dm^3^). In the case of the Folin–Ciocalteu method, the antioxidant activity was expressed in mmol GA/dm^3^.

In the next stage of the compound characterized by the highest degree of free radical scavenging DPPH (compound **8**), penetration through the pig skin and its accumulation in the skin were evaluated. For comparison, penetration studies of betulin were also carried out.

#### 4.4.1. Evaluation of Free Radical Scavenging Activity Using DPPH Method

The antioxidant activity of the tested compounds was evaluated using a modified DPPH method. The procedure was as follows: to 2.85 mL of the DPPH radical solution (at concentrations of 0.3 mmol/dm^3^) dissolved in 96% (*v*/*v*) ethanol to 0.15 mL of the tested compounds (at the concentrations of 0.226 mmol/dm^3^) was added, and then the tube was incubated for 10 min at room temperature, followed by spectrophotometric measurements at λ = 517 nm.

The antioxidant activity of the tested samples was calculated according to the following formula:

%RSA = [(A_0_ − A_p_)/A_0_] × 100% = (1 − A_p_/A_0_) × 100%, (1)

where: %RSA—antioxidant activity, A_0_—mean value of the absorbance of the ethanol solution of DPPH containing 0.15 mL of the ethanol, A_p_—mean value of absorbance of the ethanol solution of the DPPH radical containing 0.15 mL of the tested compound.

#### 4.4.2. Evaluation of Free Radical Scavenging Activity Using ABTS Method

The antioxidant activity of the tested compounds was evaluated by the ABTS method, using a solution of ABTS (at concentrations of 7 mmol/dm^3^) in an aqueous solution of potassium persulfate (at concentrations of 2.45 mmol/dm^3^) as a stock solution. The solution was incubated for 24 h, at the temperature of 4 °C and then diluted with 50% (*v/v*) methanol. The antioxidant activity was measured as follows: to 2.5 mL of a stock solution of ABTS to 0.25 mL of the tested compound (at the concentrations of 0.226 mmol/dm^3^) was added, and then the tube was incubated for 6 min at room temperature. Spectrophotometric measurements were carried out at λ = 734 nm.

The antioxidant activity was calculated from the equation:

%RSA = [(A_0_ − A_p_)/A_0_] × 100% = (1 − A_p_/A_0_) × 100%, (2)

where: %RSA—antioxidant activity, A_0_—absorbance of the stock solution ABTS containing 0.25 mL of the ethanol, A_p_—absorbance of the stock solution ABTS containing 0.25 mL of the tested compound.

#### 4.4.3. Evaluation of Total Polyphenol Content Using Folin–Ciocalteu Method

The total content of phenolic compounds found in the tested samples was evaluated using the Folin–Ciocalteu method. Two milliliters of Folin–Ciocalteu reagent in 1.8 mL of water was dissolved in a dark bottle, then the solution was incubated for 60 min (at room temperature). The antioxidant activity was measured as follows: 1.35 mL of distilled water and 1.35 mL of sodium carbonate solution (at concentrations of 0.01 mol/dm^3^) were introduced into the tube, with 0.15 mL of the prepared Folin–Ciocalteu solution and 0.15 mL of an ethanol solution containing the tested compound (at a concentration of 0.226 mmol/dm^3^), and then the tube was incubated for 15 min at room temperature, followed by spectrophotometric measurements at λ = 765 nm.

### 4.5. Skin Electrical Impedance

A fresh portion of skin from the abdomen impedance was measured using an LCR 4080 m (Conrad Electronic, Hirschau, Germany) operating in parallel mode at 120 Hz (kΩ error < 0.5%). The skin came from a local slaughterhouse. A pig skin sample was placed in a Franz diffusion cell, the permeation area of the tested compounds was 1 cm^2^.

First of all, both chambers were filled with PBS buffer, then the tips of the measuring probes were immersed in the donor chamber and the acceptor chamber. Pig skin samples with a specific impedance above 3 kU were used in the permeation of betulin and its derivative experiments. The values of pig skin electrical impedance were similar to the electrical resistance of human skin reported by others [31,33,50].

### 4.6. Skin Penetration

The effect of various alcohols (methanol, ethanol, and isopropanol) as vehicles on skin permeability for compound **8** was compared. The lag time on pig skin permeability of betulin derivative **8** was tested and compared. Preliminary studies showed that the lag time increases with increasing chain-length of the alcohol, and it is respectively: 3, 4, and 6 h. The reason for methanol application in the preliminary studies was to evaluate the effect of the chain length on the lag time of the betulin derivative. Methanol will therefore not be used in commercial vehicles for skin application. The cumulative mass of compound **8** in ethanol as a vehicle, which penetrated into the acceptor fluid was observed after 4 h of experimentation. Therefore, in this study, the penetration through the pig skin and its accumulation in the skin of ethanol vehicles containing compounds **1** and **8** (at the concentrations of 0.226 mmol/dm^3^) were evaluated.

Betulin derivatives generally have greater antioxidant activities than pure betulin [51,52,53,54]. These compounds may play an important role in the regeneration of the epidermis and the underlying layers [55,56,57]. Therefore, the assessment of skin penetration of betulin derivatives is important. Penetration of these derivatives may vary depending on their structure and lipophilicity [36,37,58,59,60,61].

The skin penetration studies were conducted in a Franz diffusion cell (SES GmbH Analyze Systeme, Germany) consisting of a 2 mL donor chamber and 8 mL acceptor chamber, which was kept at a constant temperature of 37 ± 0.5 °C with the VEB MLW Prüfgeräte-Werk type 3280 thermostat. Porcine skin was used for the study due to its permeability properties being similar to human skin. The permeation area was 1 cm^2^ [62,63].

Before starting the test, a fresh portion of the abdominal skin (with a thickness of 0.5 mm) was washed several times with PBS solution, moreover the Franz diffusion cells were allowed to equilibrate at 37 °C for 15 min. In the next stage, the skin was placed in Franz diffusion cells, and the integrity of the skin was checked one hour after its installation [5,31,47].

Ethanol solutions of betulin and its derivative **8** (at a concentration of 0.226 mmol/dm^3^) were placed in the donor chamber, then the chamber was closed with a plastic stopper to prevent excessive evaporation of the tested compounds. The process was carried out for 24 h, and aliquots of 0.3 mL of the solution in the acceptor chamber were taken at specified intervals (30 min, 1 h, 2 h, 3 h, 4 h, 5 h, 8 h, and 24 h), and then supplemented with a fresh portion of the buffer of the same pH. The samples obtained in this way were analyzed by HPLC, and then after the completion of the permeation experiment, the skin was extracted to estimate the residual amount of tested active ingredients accumulated in it.

### 4.7. Skin Extraction

To estimate the residual amount of betulin and its derivative accumulated in the skin, its extraction was carried out. The Franz diffusion chambers (*ppkt 4.5. Skin penetration*) were dismantled and the skin surface was washed three times with an aqueous solution of sodium lauryl sulfate (at a concentration of 0.5% w/w) to rinse off the excess of these compounds.

A patch (1 cm^2^ diffusion surface) was cut from the skin prepared in this way, dried at room temperature, and then weighed and cut into smaller pieces. Then, 2 mL of concentrated methanol was added, and extraction was carried out for 24 h at 4 °C. After 24 h of incubation, the skin was homogenized (for 3 min) using a homogenizer (IKA^®^T18 digital ULTRA TURRAX, Germany). The obtained extracts were then centrifuged at 3500 rpm for 5 min. The supernatant was analyzed by HPLC to determine the content of active ingredients.

The cumulative mass of active substance (µg) permeating into the receptor chamber was calculated based on the concentrations of compounds in the receptor fluid determined by HPLC. The accumulation of compounds in the skin was calculated by applying the amount of compound obtained after skin extraction; the results are given in μg/cm^2^ of skin [13,31].

### 4.8. HPLC Analysis

The content of the test compounds which penetrated into the acceptor fluid during the 24 h experiment and the amounts of extracted active ingredients which accumulated in the skin were determined using the high-performance liquid chromatography (HPLC) method, with the HPLC system from Knauer with a UV detector (Berlin, Germany).

The samples were separated on a 125 × 4 mm column containing Hyperisil ODS; particle size 5 µm. The flow rate of the mobile phase consisting of acetonitrile, water, and MeOH (28:64:8, by vol) was 1 mL/min. Twenty microliters of each analyzed sample were injected onto the column. The detection wavelength was 210 nm. Injections were repeated at least three times for each sample and the results were averaged. The concentration of betulin and its derivative was calculated based on the peak area measurements using the calibration curve method.

### 4.9. Statistical Analysis

The skin permeation results of betulin and its derivative and the accumulation of these compounds in the skin are presented as the mean ± standard deviation (SD). Statistical calculations were made using Statistica 13 PL software (StatSoft, Polska) and the Tukey post-hoc test. The results were evaluated using a one-way analysis of variance (ANOVA). Probabilities *p* < 0.05 were considered statistically significant.

## 5. Conclusions

During the research, betulin (compound **1**) and its derivatives (compounds **2**–**11**) were obtained and characterized. The penetration of compounds **1** and **8** from the vehicle through the skin was observed (under physiological conditions 37 ℃, pH = 7.4), and it was demonstrated that the betulin derivative penetrates through biological membranes more readily than the initial betulin. Moreover, betulin and its derivative **8** also accumulated in the skin.

The obtained results indicate the possibility of using these compounds as an ingredient in cosmetics and pharmaceutics applied to the skin. Betulin and some betulin derivatives inhibit the formation of reactive oxygen species (ROS) [64], therefore vehicles containing these compounds look promising as antioxidant delivery systems.

The biological activity of betulin is well documented. Furthermore, the vehicles are approved for use in active substances delivery systems. The use of such a solvent as a vehicle in biological systems enables a controlled release of betulin and its derivative **8**, which is dictated by the penetration rate of the vehicles.

## Figures and Tables

**Table 1:** Partition coefficient values calculated by theoretical method, in the MestReNova program.

Compounds	Formula	Molecular Weight[Da]	*log* P
**1**	C_30_H_50_O_2_	442.73	5.642
**2**	C_30_H_46_O_2_	438.7	5.372
**3**	C_30_H_46_O_3_	454.7	4.777
**4**	C_32_H_52_O_3_	484.77	6.430
**5**	C_34_H_54_O_4_	526.8	7.000
**6**	C_34_H_53_BrO_4_	605.7	6.997
**7**	C_32_H_54_O_3_	486.78	6.779
**8**	C_32_H_52_O_3_	484.77	6.304
**9**	C_32_H_52_O_4_	500.76	6.613
**10**	C_30_H_54_O_3_	462.76	5.006
**11**	C_46_H_72_O_3_Si	701.16	9.684

**Table 2:** Antioxidant activity of betulin and its derivatives.

Compounds	* Antioxidant Activity (DPPH Method):	
	% RSA	mmol TE/dm^3^
**1**	2.81 ± 0.007	0.087 ± 0.003
**2**	1.50 ± 0.007	0.079 ± 0.011
**3**	7.04 ± 0.007	0.091 ± 0.001
**4**	n.a.	n.a.
**5**	n.a.	n.a.
**6**	14.78 ± 0.014	0.100 ± 0.002
**7**	2.80 ± 0.012	0.087 ± 0.001
**8**	61.83 ± 0.004	0.436 ± 0.005
**9**	14.11 ± 0.013	0.099 ± 0.002
**10**	4.84 ± 0.011	0.089 ± 0.001
**11**	12.68 ± 0.013	0.098 ± 0.001
	*Antioxidant Activity (ABTS method):	
	% RSA	mmol TE/dm^3^
**1**	n.a.	n.a.
**2**	n.a.	n.a.
**3**	n.a.	n.a.
**4**	n.a.	n.a.
**5**	n.a.	n.a.
**6**	n.a.	n.a.
**7**	n.a.	n.a.
**8**	12.79 ± 0.005	0.237 ± 0.002
**9**	n.a.	n.a.
**10**	n.a.	n.a.
**11**	5.65 ± 0.015	0.105 ± 0.002
	*Antioxidant Activity (Folin–Ciocalteu Method):	
		mmol TE/dm^3^
**1**	-	n.a.
**2**	-	n.a.
**3**	-	0.847 ± 0.037
**4**	-	n.a.
**5**	-	n.a.
**6**	-	0.367 ± 0.081
**7**	-	n.a.
**8**	-	0.050 ± 0.000
**9**	-	0.413 ± 0.015
**10**	-	n.a.
**11**	-	n.a.

n.a.—no activity, * Mean ± SD (*n* = 3).

**Table 3:** Permeation of active substances through skin and the amounts of extracted active ingredients accumulated in it.

Compounds	The Cumulative Mass of Substance after 24 h of Permeation Test: (µg)	Skin Accumulation of Substance: (µg/cm^2^ Skin)
* Betulin	14.27 ± 2.20 a	135.71 ± 9.11 a
* Betulin derivative **8**	21.41 ± 2.10 b	104.06 ± 15.79 b

* Mean ± SD (*n* = 3), a, b—values are significantly different between analyzed vehicles (*P* = 0.001).

## Data Availability

The data presented in this study are available in this article.

## Supplementary Materials

The following are available online, ^1^H NMR and ^13^C NMR spectra of compound 1–11.

## Sample Availability

Samples of the compounds are available from the authors.
