# Supplementary material for: Enhancement of the Antioxidant and Skin Permeation Properties of Betulin and Its Derivatives
Source: Molecules. 2021 Jun 5;26(11):3435. doi: 10.3390/molecules26113435 (PMC8201114; doi:10.3390/molecules26113435)
Supplement: Supplementary file 1 [file molecules-26-03435-s001.zip › molecules-1244433-supplementary.pdf]

Supplementary material

Enhancement of the Antioxidant and Skin Permeation Properties of Betulin and its Derivatives

Andrzej Günther [1,*], Edyta Makuch [1], Anna Nowak [2], Wiktoria Duchnik [2], Łukasz Kucharski [2], Robert Pełech [1] and Adam Klimowicz [2]

[1] Faculty of Chemical Technology and Engineering, Department of Chemical Organic Technology and Polymeric Materials, West Pomeranian University of Technology, Szczecin, PL-70322 Szczecin, Poland; emakuch@zut.edu.pl E.M.); rpełech@zut.edu.pl (R.P.)

[2] Department of Cosmetic and Pharmaceutical Chemistry, Pomeranian Medical University in Szczecin, PL-70111 Szczecin, Poland; anowak@pum.edu.pl (A.N.); wduchnik@pum.edu.pl (W.D.); lukasz.kucharski@pum.edu.pl (Ł.K.); adam.klimowicz@pum.edu.pl (A.K.)

* Correspondence: andrzej.gunther@zut.edu.pl;

Table of contents

1H NMR and 13C NMR spectra of compound (1) S 3

1H NMR and 13C NMR spectra of compound (2) S 4

1H NMR and 13C NMR spectra of compound (3) S 5

1H NMR and 13C NMR spectra of compound (4) S 6

1H NMR and 13C NMR spectra of compound (5) S 7

1H NMR and 13C NMR spectra of compound (6) S 8

1H NMR and 13C NMR spectra of compound (7) S 9

1H NMR and 13C NMR spectra of compound (8) S 10

1H NMR and 13C NMR spectra of compound (9) S 11

1H NMR and 13C NMR spectra of compound (10) S 12

1H NMR and 13C NMR spectra of compound (11) S 13

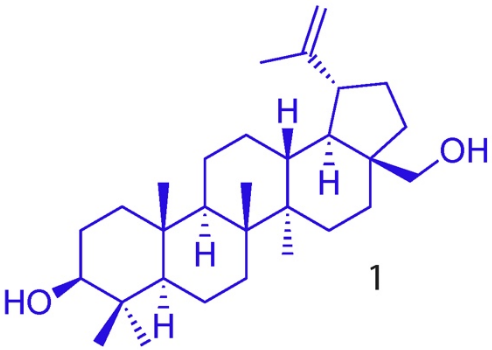
HO
OH
H

1

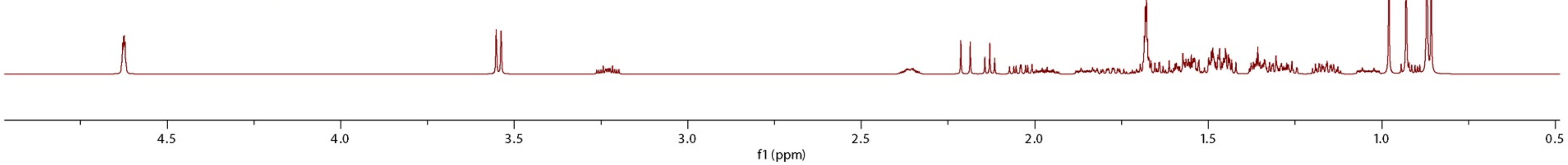
4.5
4.0
3.5
3.0
2.5
2.0
1.5
1.0
0.5
f1 (ppm)

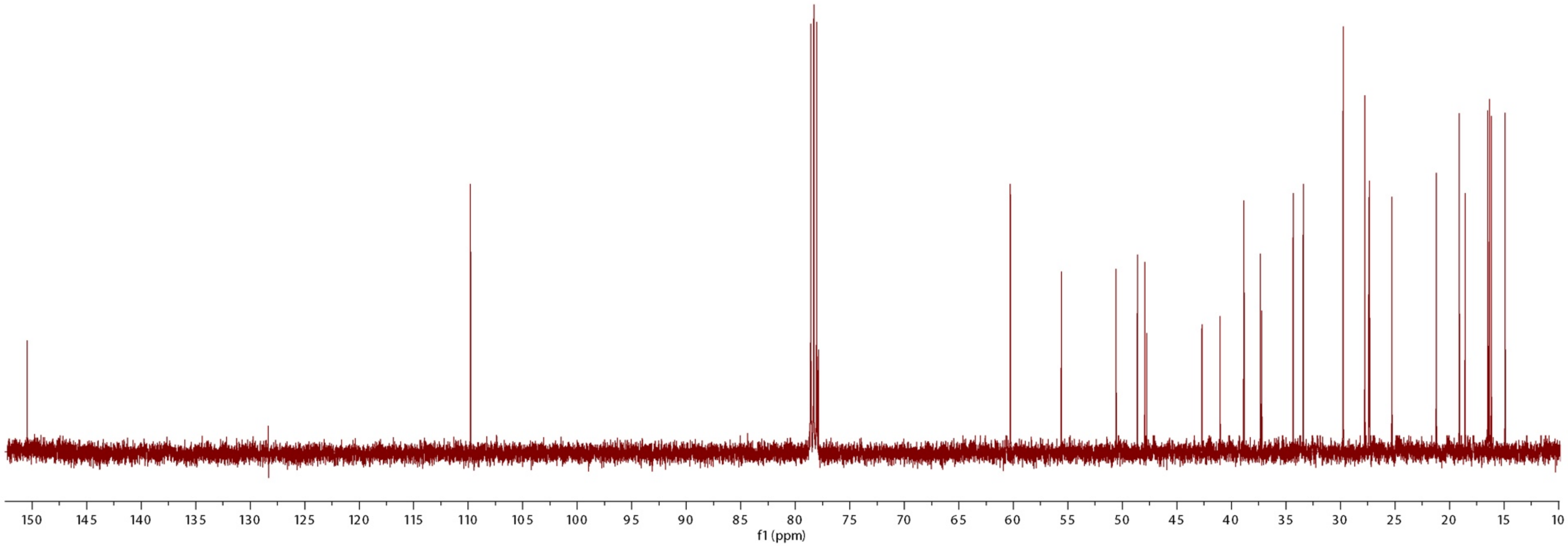
150
145
140
135
130
125
120
115
110
105
100
95
90
85
80
75
70
65
60
55
50
45
40
35
30
25
20
15
10
f1 (ppm)

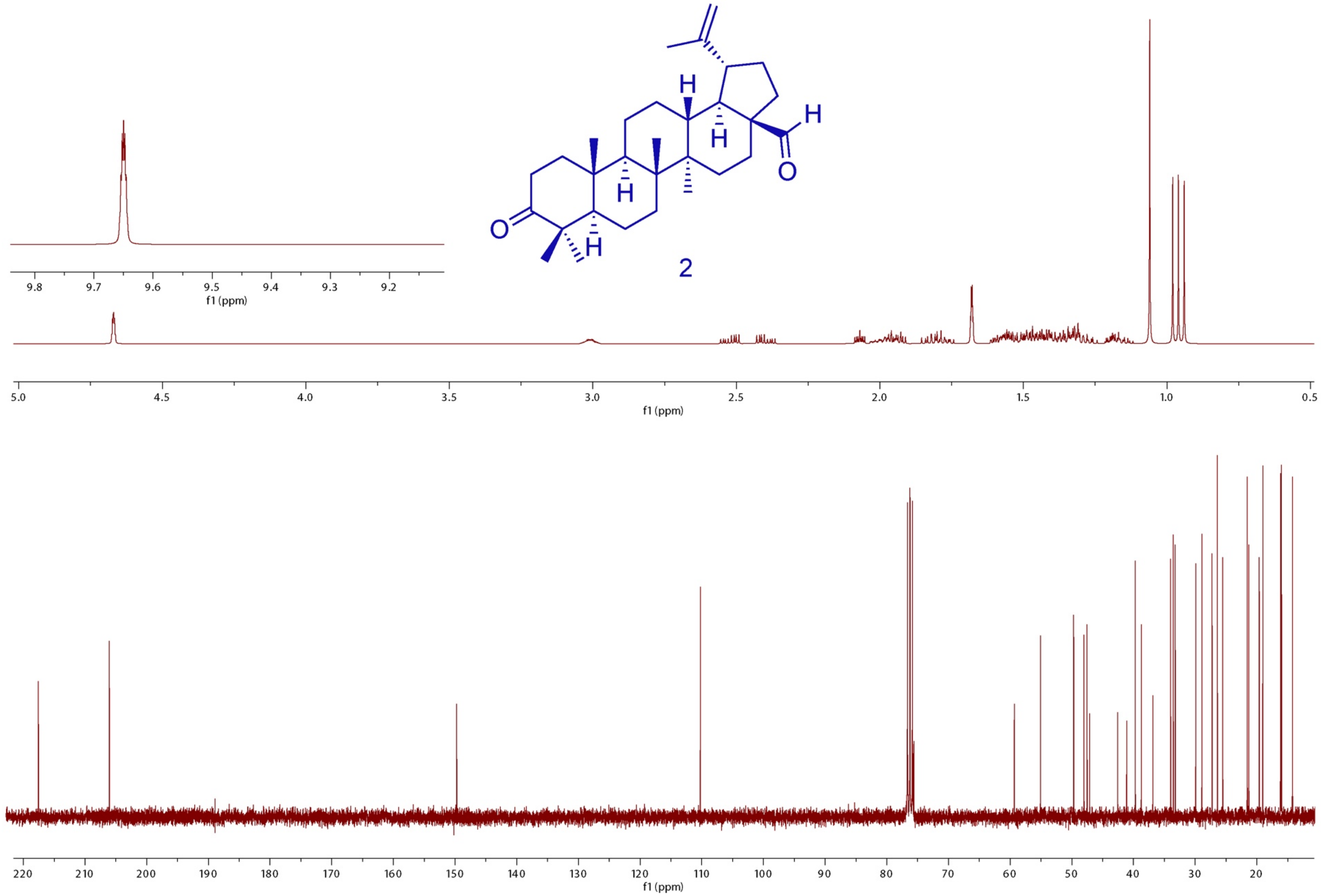
2
9.8 9.7 9.6 9.5 9.4 9.3 9.2
f1 (ppm)
5.0 4.5 4.0 3.5 3.0 2.5 2.0 1.5 1.0 0.5
f1 (ppm)
220 210 200 190 180 170 160 150 140 130 120 110 100 90 80 70 60 50 40 30 20
f1 (ppm)

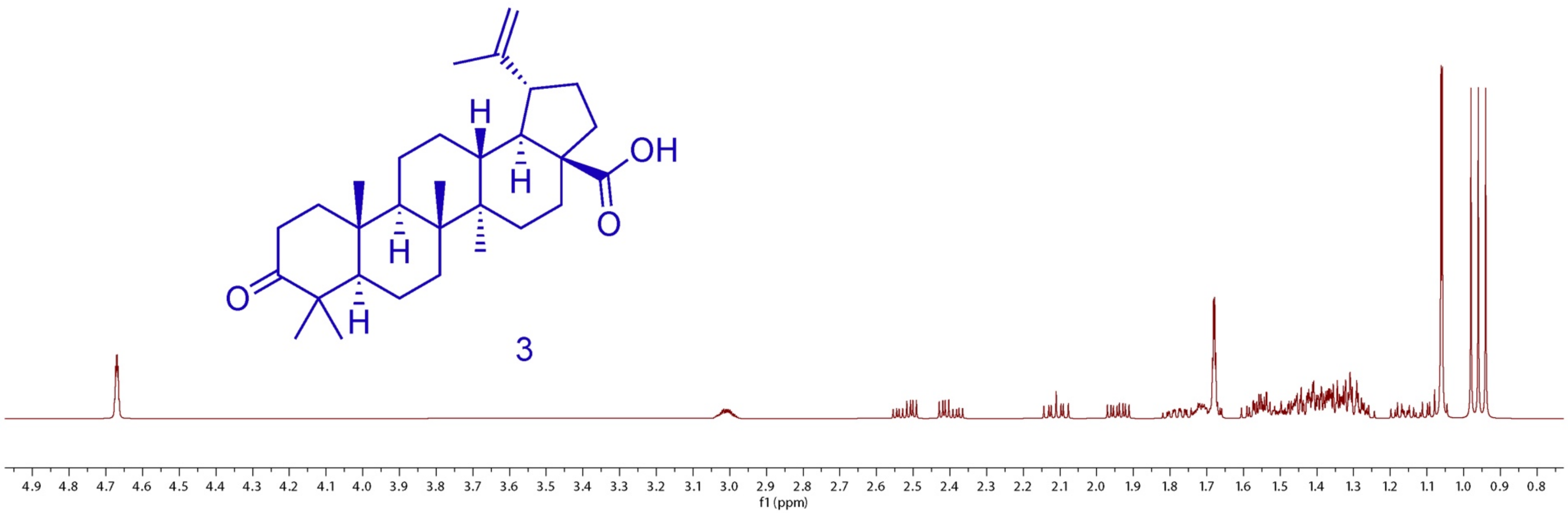

OH
O
O
H
H
H
H
3
4.9 4.8 4.7 4.6 4.5 4.4 4.3 4.2 4.1 4.0 3.9 3.8 3.7 3.6 3.5 3.4 3.3 3.2 3.1 3.0 2.9 2.8 2.7 2.6 2.5 2.4 2.3 2.2 2.1 2.0 1.9 1.8 1.7 1.6 1.5 1.4 1.3 1.2 1.1 1.0 0.9 0.8
f1 (ppm)

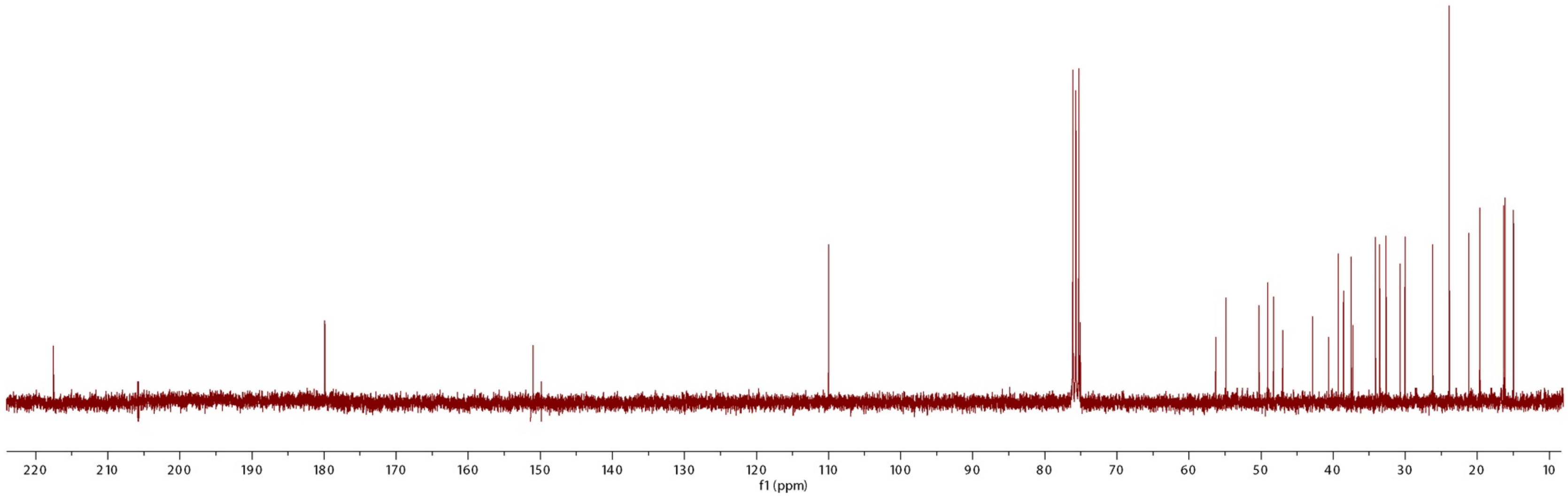

220 210 200 190 180 170 160 150 140 130 120 110 100 90 80 70 60 50 40 30 20 10
f1 (ppm)

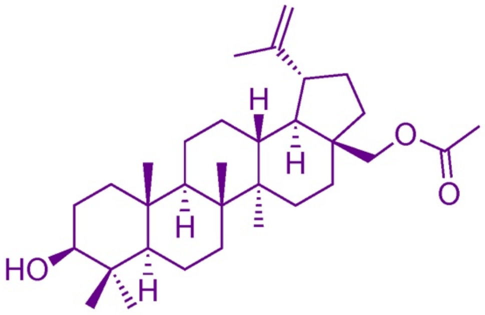
HO
H
H
H
H
O
O

4

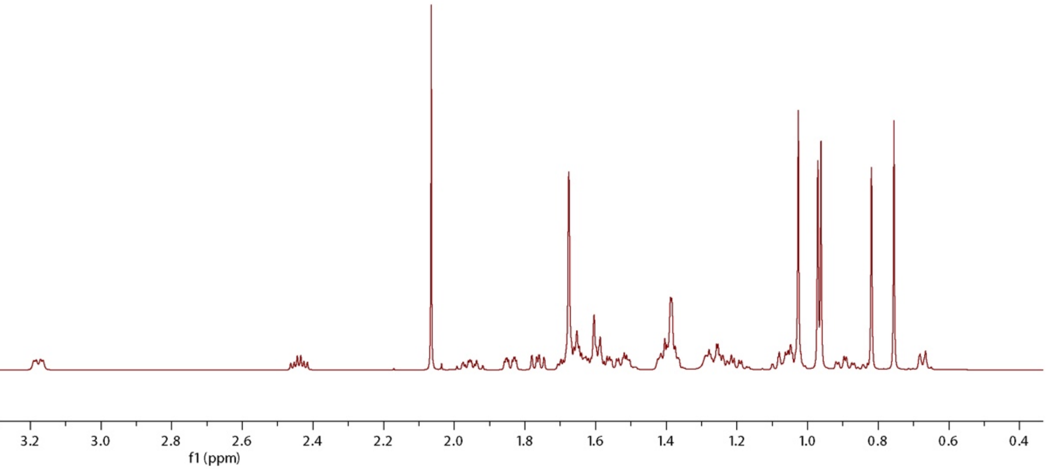
5.0 4.8 4.6 4.4 4.2 4.0 3.8 3.6 3.4 3.2 3.0 2.8 2.6 2.4 2.2 2.0 1.8 1.6 1.4 1.2 1.0 0.8 0.6 0.4
f1 (ppm)

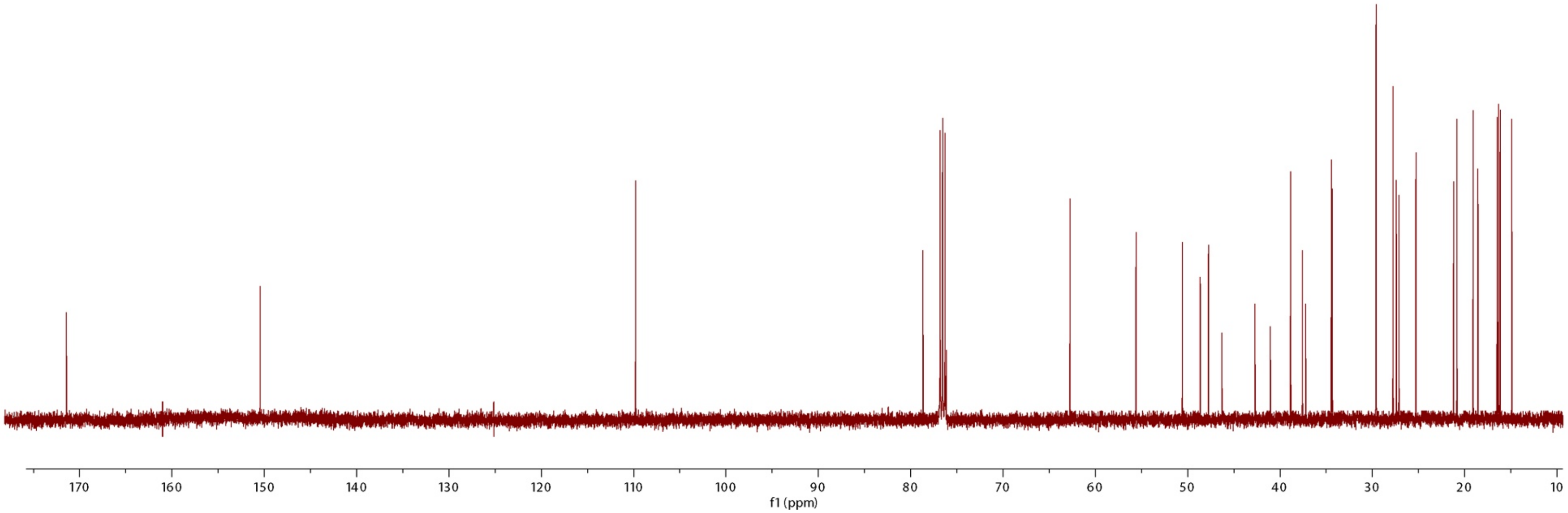
170 160 150 140 130 120 110 100 90 80 70 60 50 40 30 20 10
f1 (ppm)

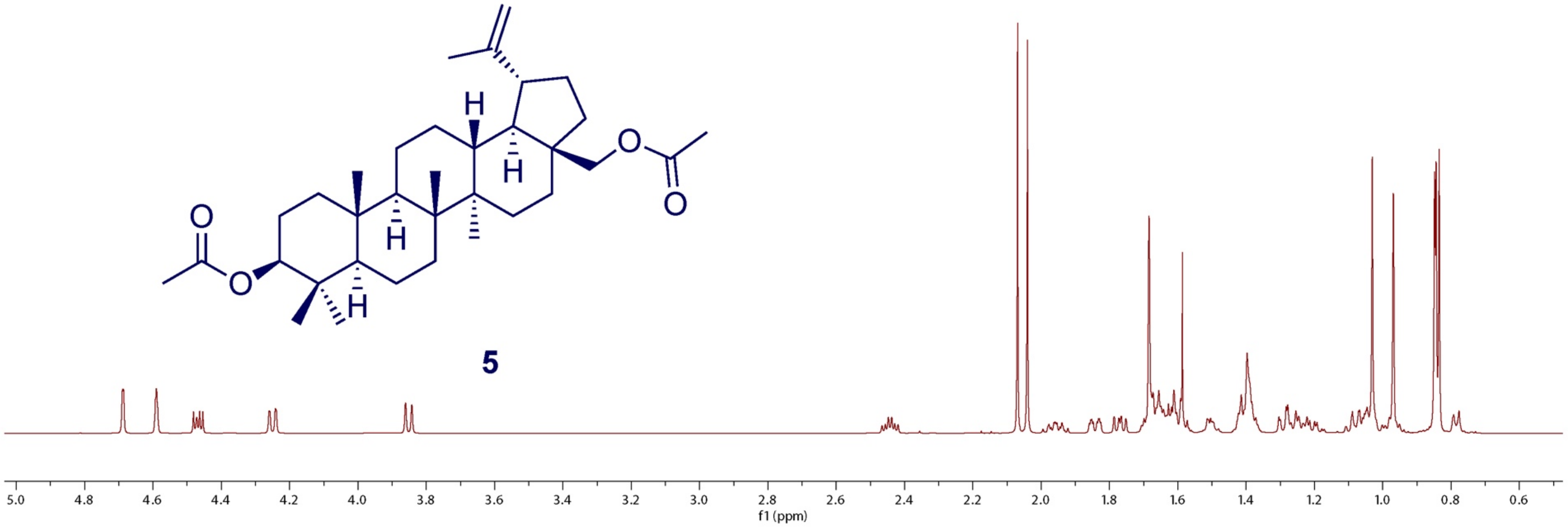
5.0
4.8
4.6
4.4
4.2
4.0
3.8
3.6
3.4
3.2
3.0
2.8
2.6
2.4
2.2
2.0
1.8
1.6
1.4
1.2
1.0
0.8
0.6
f1 (ppm)

5

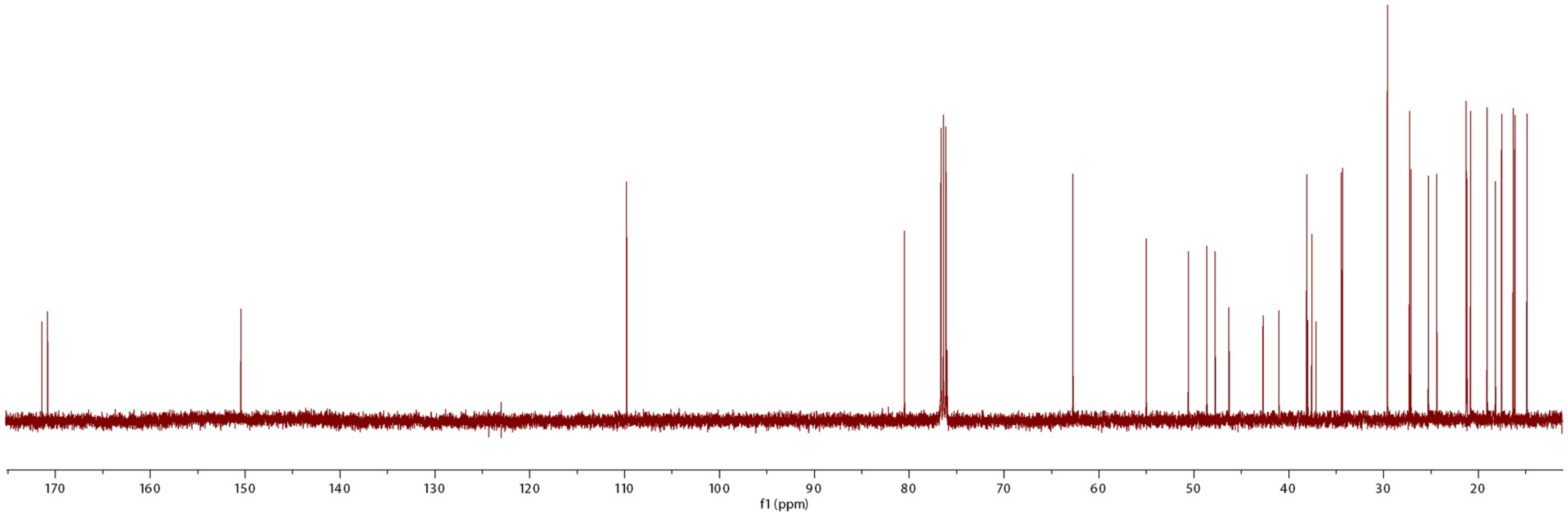
170
160
150
140
130
120
110
100
90
80
70
60
50
40
30
20
f1 (ppm)

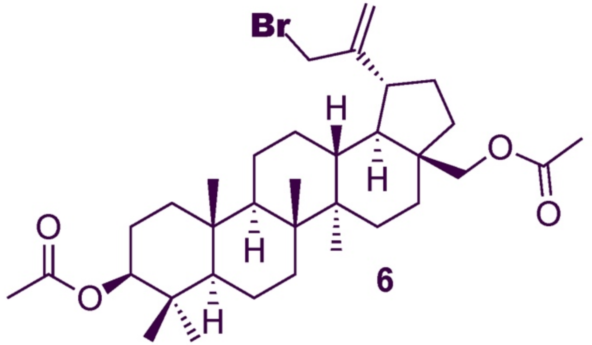
Br
H
H
H
H
O
O
O
O

6

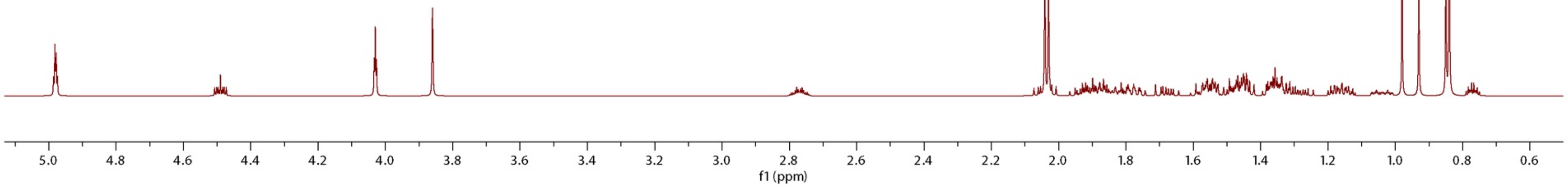
5.0 4.8 4.6 4.4 4.2 4.0 3.8 3.6 3.4 3.2 3.0 2.8 2.6 2.4 2.2 2.0 1.8 1.6 1.4 1.2 1.0 0.8 0.6
f1 (ppm)

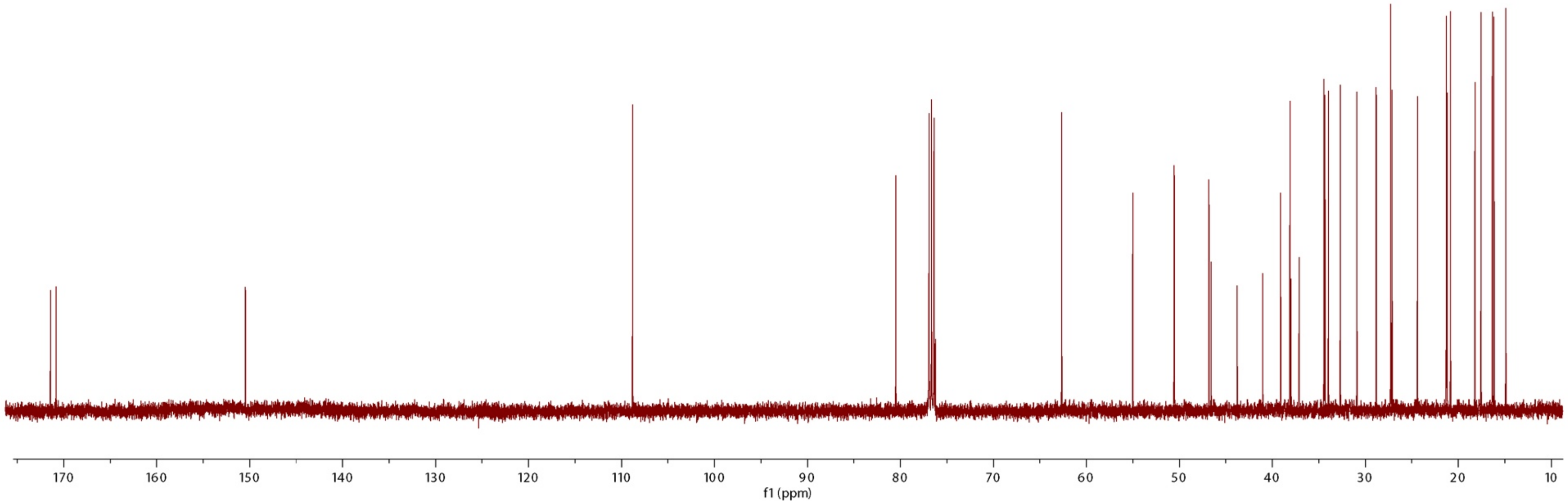
170 160 150 140 130 120 110 100 90 80 70 60 50 40 30 20 10
f1 (ppm)

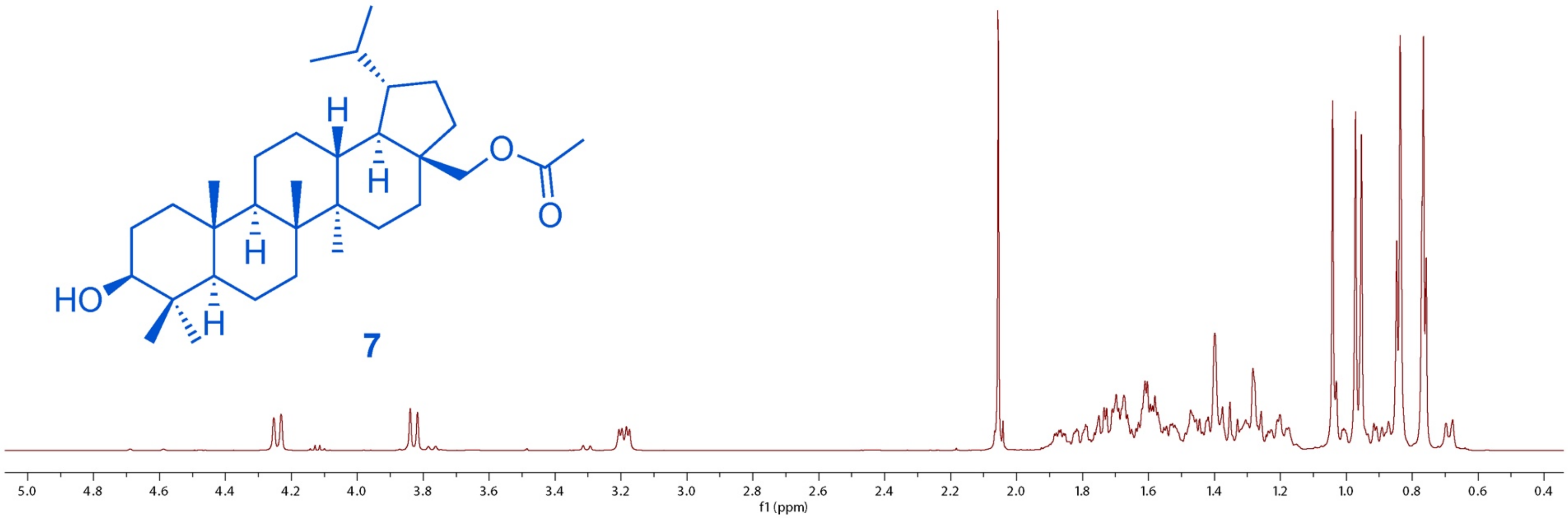
HO
H
O
O
7
5.0 4.8 4.6 4.4 4.2 4.0 3.8 3.6 3.4 3.2 3.0 2.8 2.6 2.4 2.2 2.0 1.8 1.6 1.4 1.2 1.0 0.8 0.6 0.4
f1 (ppm)

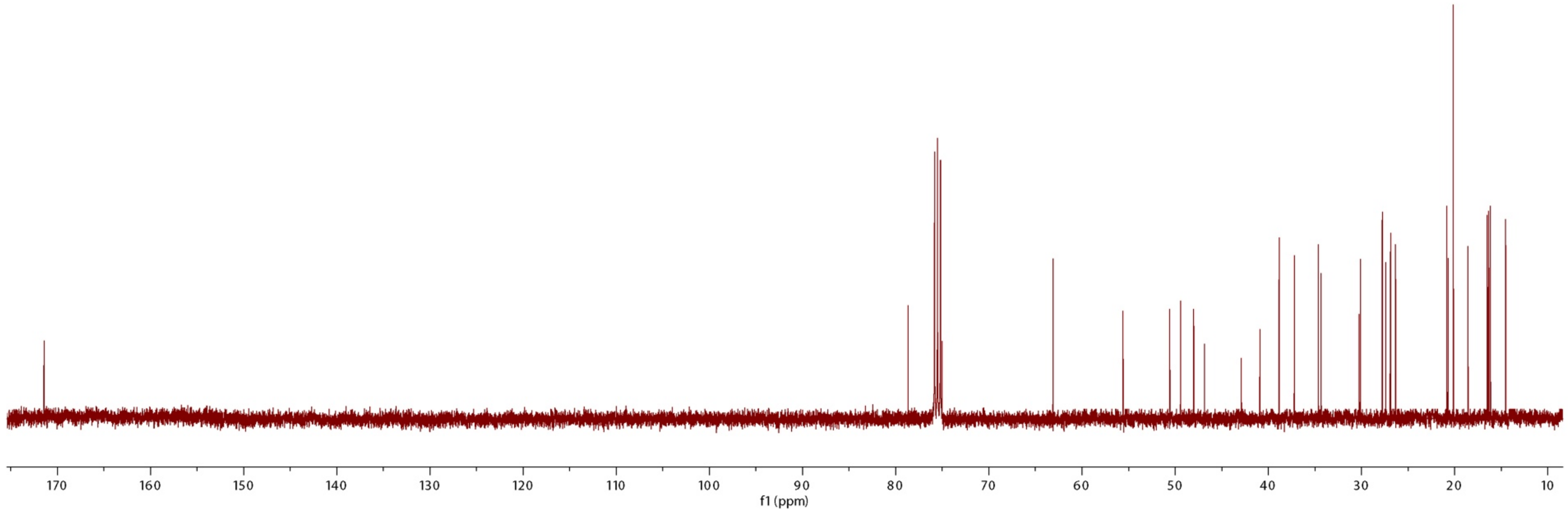
170 160 150 140 130 120 110 100 90 80 70 60 50 40 30 20 10
f1 (ppm)

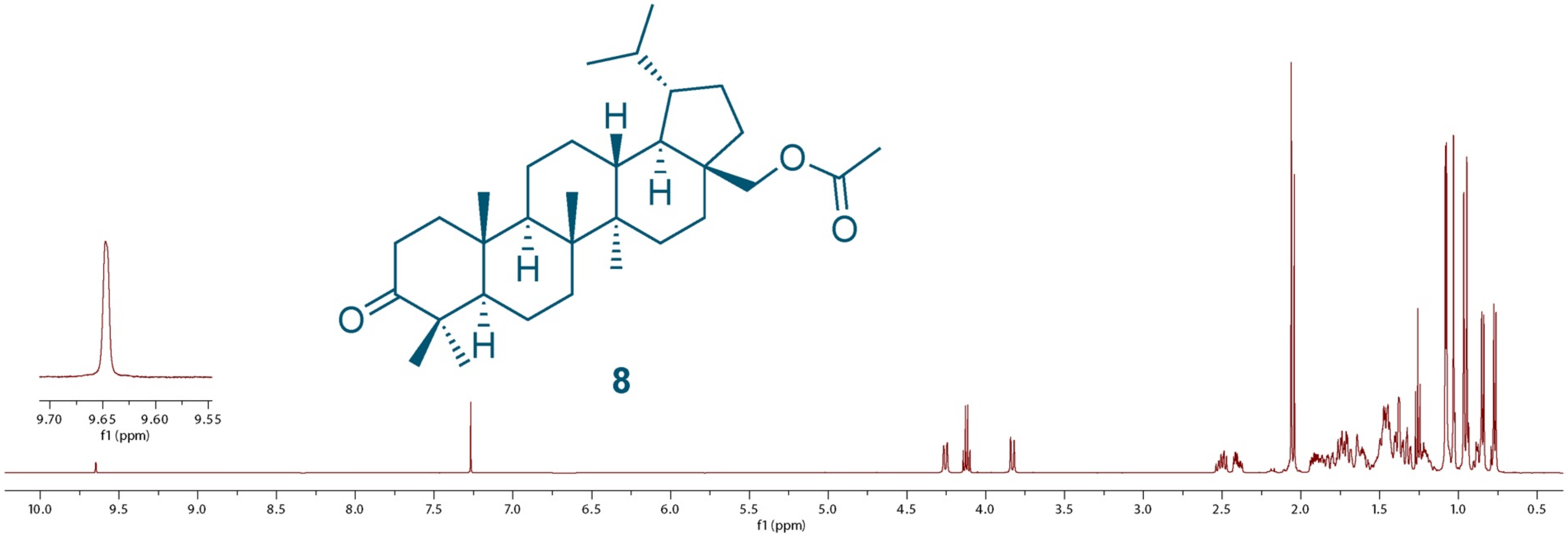

8

9.70 9.65 9.60 9.55
f1 (ppm)
10.0 9.5 9.0 8.5 8.0 7.5 7.0 6.5 6.0 5.5 5.0 4.5 4.0 3.5 3.0 2.5 2.0 1.5 1.0 0.5
f1 (ppm)

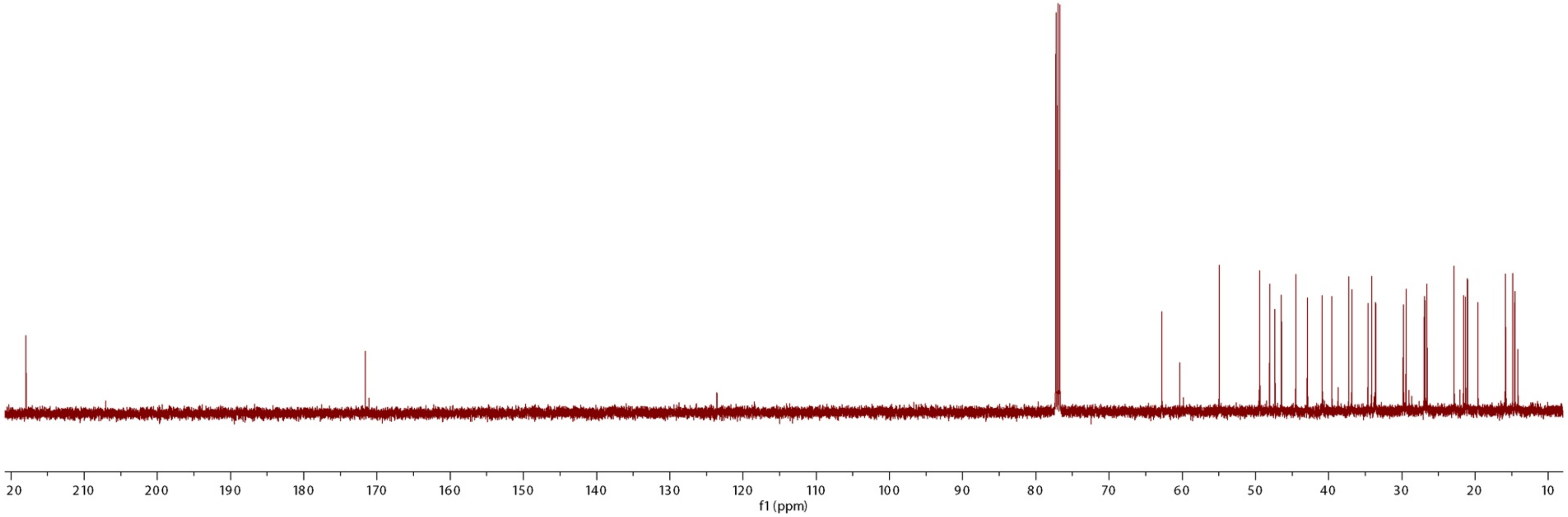

20 210 200 190 180 170 160 150 140 130 120 110 100 90 80 70 60 50 40 30 20 10
f1 (ppm)

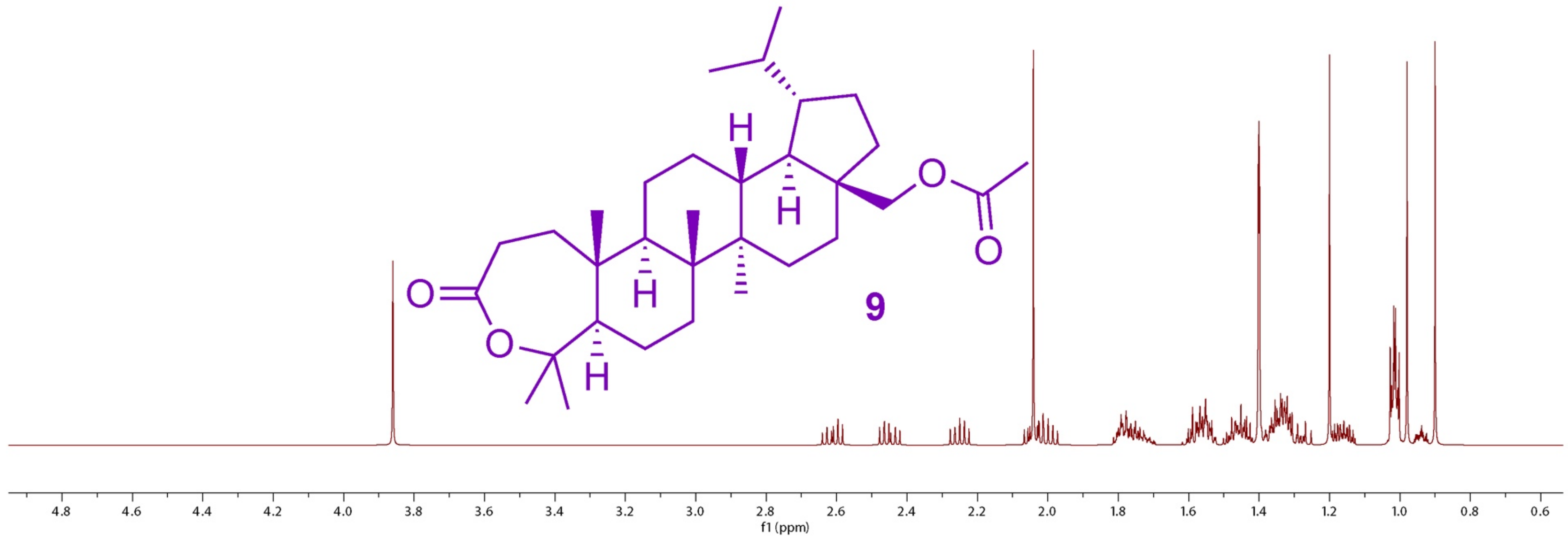
9
4.8
4.6
4.4
4.2
4.0
3.8
3.6
3.4
3.2
3.0
2.8
2.6
2.4
2.2
2.0
1.8
1.6
1.4
1.2
1.0
0.8
0.6
f1 (ppm)

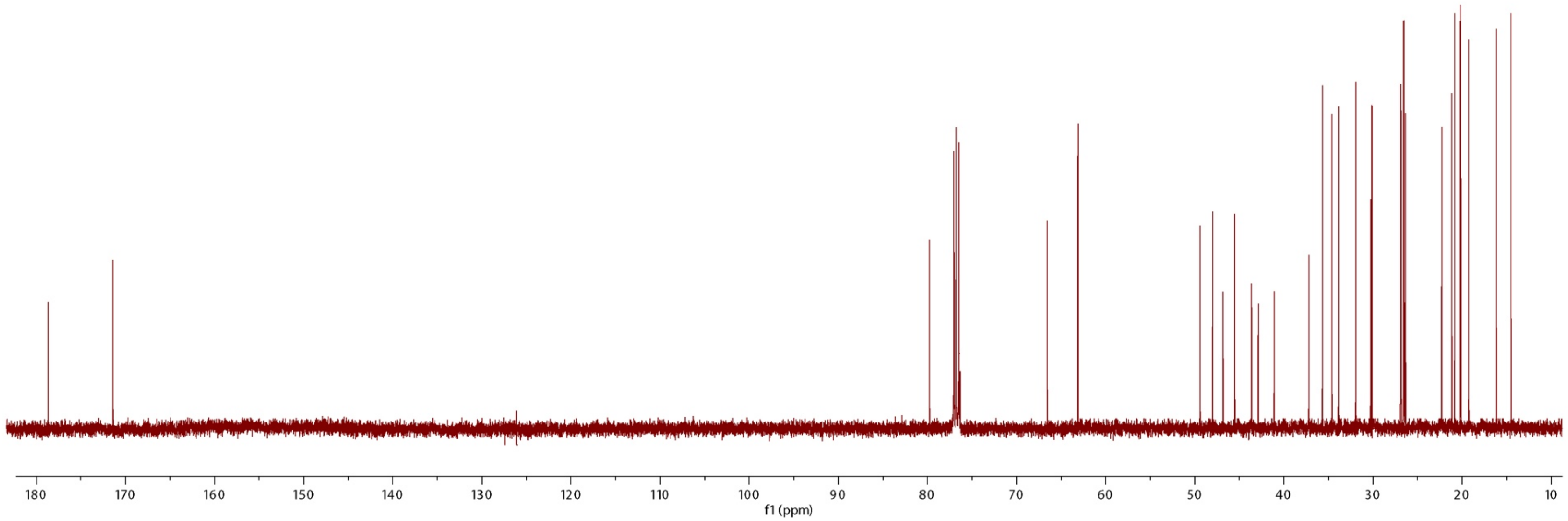
180
170
160
150
140
130
120
110
100
90
80
70
60
50
40
30
20
10
f1 (ppm)

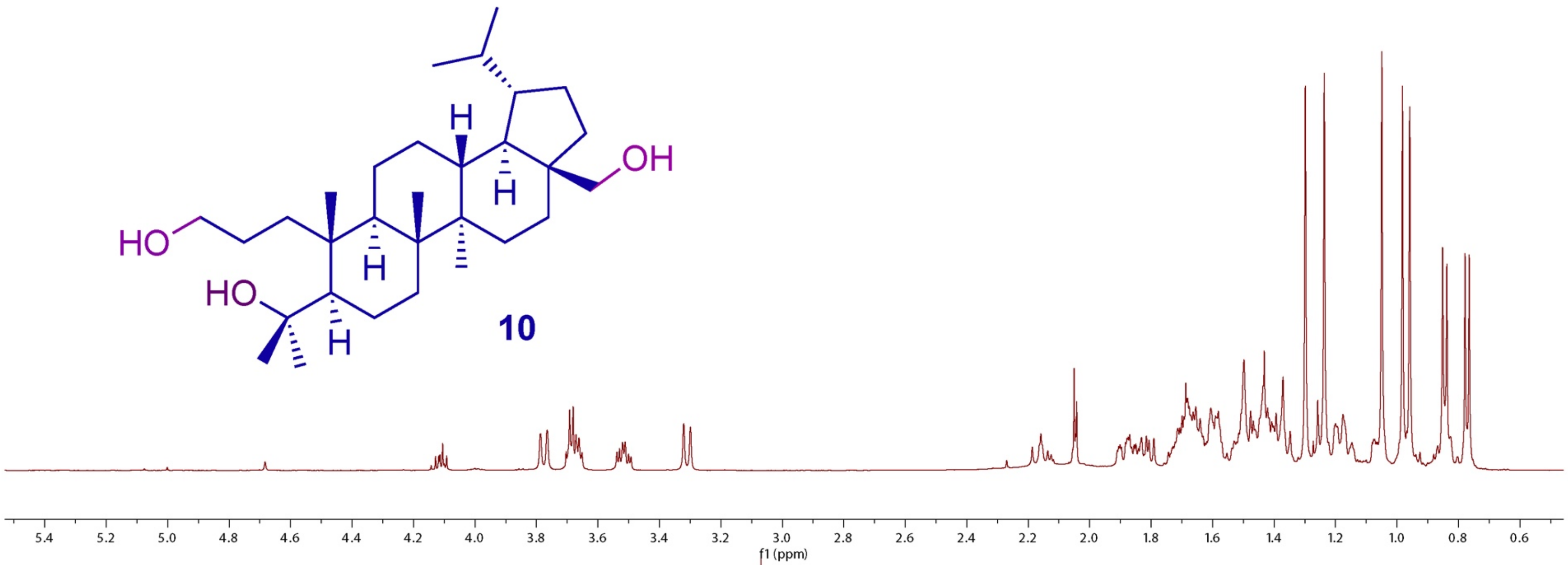
HO
HO
OH
H
H
H
H
10
5.4 5.2 5.0 4.8 4.6 4.4 4.2 4.0 3.8 3.6 3.4 3.2 3.0 2.8 2.6 2.4 2.2 2.0 1.8 1.6 1.4 1.2 1.0 0.8 0.6
f1 (ppm)

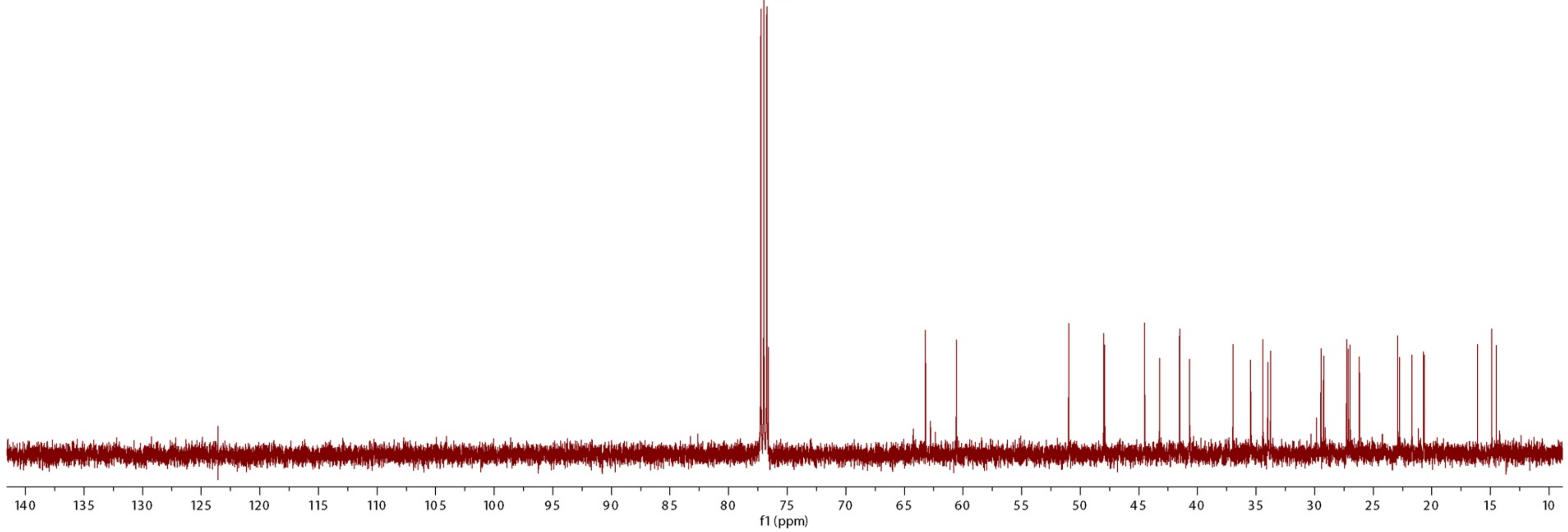
140 135 130 125 120 115 110 105 100 95 90 85 80 75 70 65 60 55 50 45 40 35 30 25 20 15 10
f1 (ppm)

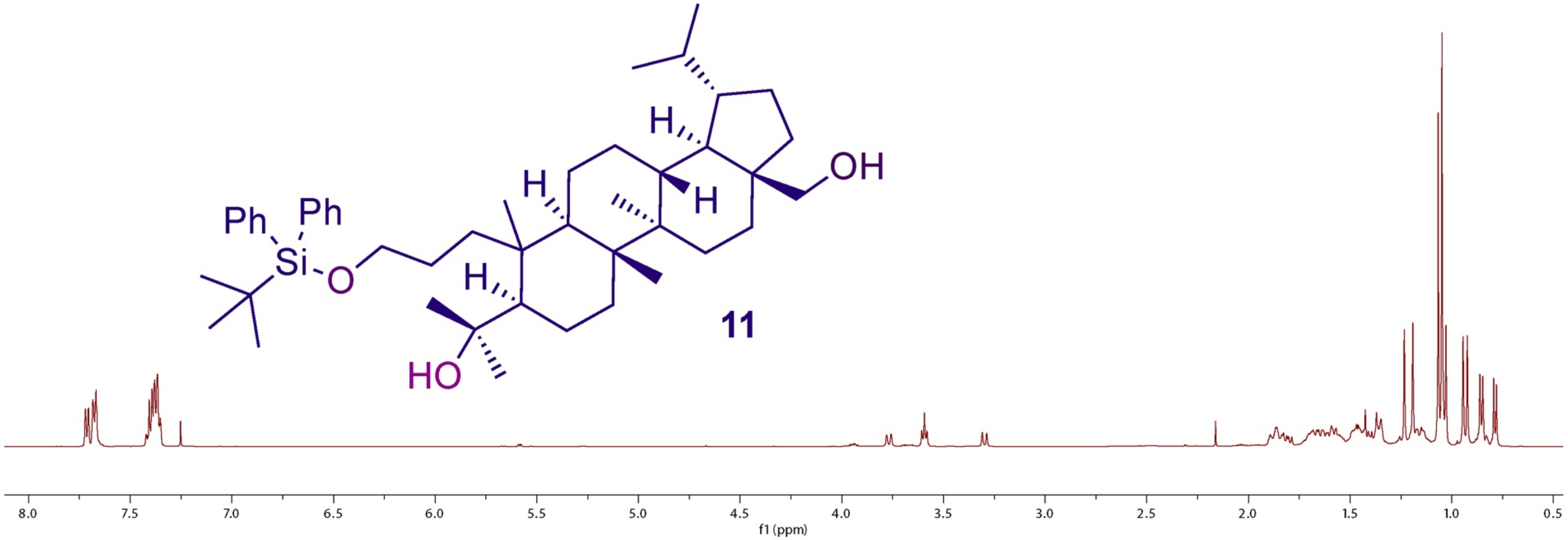

Ph
Ph
Si
O
OH
HO
H
11
8.0 7.5 7.0 6.5 6.0 5.5 5.0 4.5 4.0 3.5 3.0 2.5 2.0 1.5 1.0 0.5
f1 (ppm)

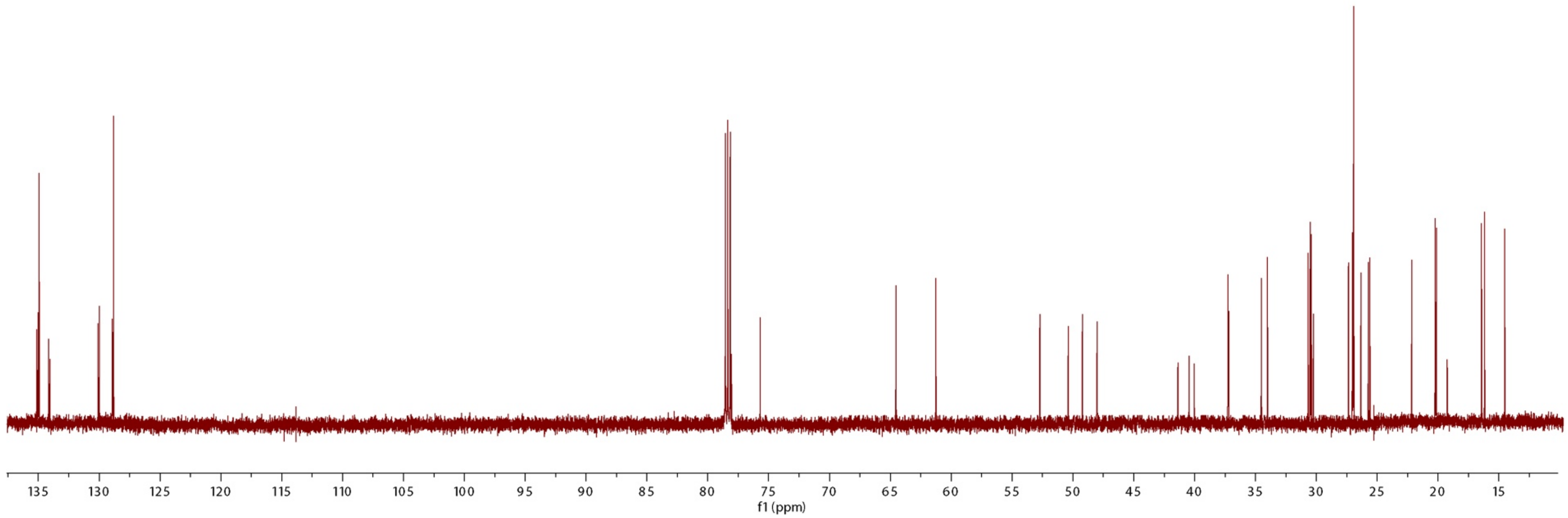

135 130 125 120 115 110 105 100 95 90 85 80 75 70 65 60 55 50 45 40 35 30 25 20 15
f1 (ppm)
